# Reports of Plant-Derived Nanoparticles for Prostate Cancer Therapy

**DOI:** 10.3390/plants12091870

**Published:** 2023-05-03

**Authors:** Abdulrahman M. Elbagory, Rodney Hull, Mervin Meyer, Zodwa Dlamini

**Affiliations:** 1 Department of Science and Innovation (DSI)/Mintek Nanotechnology Innovation Centre (NIC), Biolabels Research Node, Department of Biotechnology, University of the Western Cape, Cape Town, Private Bag X17, Bellville 7535, South Africa; 3376881@myuwc.ac.za; 2 SAMRC Precision Oncology Research Unit (PORU), DSI/NRF SARChI Chair in Precision Oncology and Cancer Prevention (POCP), Pan African Cancer Research Institute (PACRI), University of Pretoria, Hatfield 0028, South Africa; rodney.hull@up.ac.za

**Keywords:** green nanotechnology, phytochemicals, plant-derived nanoparticles, EGCG nanoparticles, prostate cancer

## Abstract

Plants have demonstrated potential in providing various types of phytomedicines with chemopreventive properties that can combat prostate cancer. However, despite their promising in vitro activity, the incorporation of these phytochemicals into the market as anticancer agents has been hindered by their poor bioavailability, mainly due to their inadequate aqueous solubility, chemical instability, and unsatisfactory circulation time. To overcome these drawbacks, it has been suggested that the incorporation of phytochemicals as nanoparticles can offer a solution. The use of plant-based chemicals can also improve the biocompatibility of the formulated nanoparticles by avoiding the use of certain hazardous chemicals in the synthesis, leading to decreased toxicity in vivo. Moreover, in some cases, phytochemicals can act as targeting agents to tumour sites. This review will focus on and summarize the following points: the different types of nanoparticles that contain individual phytochemicals or plant extracts in their design with the aim of improving the bioavailability of the phytochemicals; the therapeutic evaluation of these nanoparticles against prostate cancer both in vitro and in vivo and the reported mode of action and the different types of anticancer experiments used; how the phytochemicals can also improve the targeting effects of these nanoparticles in some instances; and the potential toxicity of these nanoparticles.

## 1. Introduction

Prostate cancer (PC) is a prevalent malignancy that is among the top five leading causes of cancer-related deaths worldwide [1]. In 2019, approximately 174,650 new PC cases were diagnosed, accounting for 20% of all new cancer cases in men [2]. Chemotherapy is a commonly used treatment for PC, which aims to eliminate rapidly dividing cancer cells. However, drug resistance and adverse side effects often arise from the use of chemotherapeutics drugs such as doxetaxel, carbazetaxel, mitoxantrone, doxorubicin, etc. [3]. The use of androgen deprivation therapies can also have detrimental effects on the skeletal metabolism [4]. Hence, there is a pressing need to explore new medications that can effectively treat PC with minimal side effects.

Nanoparticles (NPs) hold promise as an effective treatment option for PC owing to their ability to passively target solid tumours by exploiting the enhanced permeability and retention effect of tumours [5]. NPs, due to their small size, can bypass non-targeted therapy-associated side effects after systemic administration, while also increasing the efficiency of the loaded anticancer agents [6]. Moreover, NPs can actively target tissues when coupled with receptor targeting molecules [6]. This can be helpful in reaching PC tissue by targeting several upregulated biomarkers which were identified in PC cells. For example, prostate-specific membrane antigen (PSMA) is one of these biomarkers, which is overexpressed in the epithelium of malignant prostates at levels a thousand times more than the expression observed in other normal tissues [7]. PSMA can be targeted for both therapeutic and diagnostic purposes [8]. Another example is urokinase-type plasminogen activator (uPA), which is involved in tumour invasion and metastasis [9]. uPA plays a crucial role in the process of converting inactive plasminogen into active plasmin. This, in turn, has a significant impact on various events that occur during the metastatic cascade [10]. Downregulating uPA in androgen-insensitive human prostate cancer cell lines (PC3 and Du145) implanted in mouse models was found to cause apoptosis and reduce PC metastasis [11]. Additionally, the multifunctional protein, CD44, is another major biomarker of prostate-cancer-initiating cells that is responsible for PC tumorgenicity [12]. CD44 and its isoforms have clinicopathological effects that promote tumorigenesis and help cancer stem cells maintain their stemness during tumour regeneration after therapy. These effects suggest that CD44 could serve as a target for cancer treatment or as a valuable prognostic marker [13]. Some examples of green-based nanoparticles targeting these cancer biomarkers are mentioned in this review.

Plants are rich in bioactive phytochemicals that can suppress and prevent cancer development [14]. In fact, some of the most well-known chemotherapeutics are derived from plant sources. To exemplify, *Catharanthus roseus* is the natural source of two anticancer vinca alkaloids, vinblastine and vincristine, while Paclitaxel (Taxol) was originally isolated from *Taxus brevifolia* [15]. Despite this, many of the newly discovered phytochemicals considered for anticancer treatment are characterized by poor pharmacokinetics, which limit their use in medicinal applications. For example, the ability of green tea catechins, such as Epigallocatehin Gallate (EGCG), to be absorbed through oral consumption into human plasma was discovered to be 5 to 50 times lower than the level necessary for it to exhibit biological effects in in vitro systems [16]. Additionally, the low absorption rate of curcumin by the small intestine, combined with its significant reductive and conjugative metabolism in the liver, greatly diminishes its ability to be absorbed through oral consumption [17]. Further, when administered orally, the bioavailability of berberine is low because of first-pass elimination [18]. The clinical trials of these compounds in their free forms have also shown unpromising data to support their use in cancer therapy. In a double-blind trial where green tea was standardized to contain 150 mg of EGCG twice daily, there was no notable difference in the rate of colon adenoma between the group that received green tea and the group that received the placebo [19]. Similar findings were also reported in a placebo-controlled study that showed no effect of the oral consumption of 830 mg green tea on the prostate cancer tissue [20]. Additionally, the results of a phase II study conducted on patients with metastatic castration-resistant prostate cancer clearly demonstrated that incorporating curcumin into the treatment plans did not yield effective results [21]. Research has demonstrated that incorporating phytochemicals into nanoparticles can enhance their ability to permeate through blood vessels and the gastrointestinal tract, while also improving selectivity towards tumour cells [22]. This in return leads to increased drug uptake, the inhibition of hepatic first-pass metabolism, improved drug solubility and stability, and reduced elimination via reticuloendothelial organs [22].

Furthermore, plant phytochemicals can not only be loaded onto NPs to assist their delivery to the target sites or to improve their pharmacokinetics, but also can be involved in the biosynthesis of NPs as a way of lowering the toxicity of the NPs to be suitable for biomedical applications [23]. The conventional physical and chemical methods for synthesizing NPs involve the use of toxic and hazardous chemicals such as sodium/potassium borohydrate, hydrazine, sodium citrate, and salts of tartrate, which require high energy consumption, are expensive, and involve complex techniques [24,25]. In contrast, the green biosynthesis of NPs offers an easy, convenient, scalable, and eco-friendly alternative that requires less energy [26]. Plants possess polyphenols and proteins in their extracts that can serve as alternatives to chemical reagents in reducing metal ions into lower-valence states [27]. The role of functional groups of phytochemicals in biosynthesis can be identified via several analytical techniques such as Fourier transform infrared (FTIR) [28]. In addition to isolated phytochemicals, such as EGCG and curcumin, noscapine, berberine, and eupatorin, this review will also highlight several studies that used plant extracts in the biosynthesis of NPs with reported activity against PC.

## 2. Results

Table 1 lists studies identified from the PubMed search platform that used green synthesized NPs for the treatment of PC.

### 2.1. EGCG-Based NPs

Around 30% of the dry weight of green tea contains antioxidant polyphenolic compounds, and it has been proposed that these compounds are mainly responsible for the chemotherapeutic effects of green tea [60]. Catechins are a major component of green tea polyphenols, of which EGCG is the most abundant [61]. EGCG has a polyphenolic structure (eight hydroxyl groups) that allows for electron delocalization, leading to the neutralization of ROS and nitrogen species. This is the reason why EGCG has protective effects against cancer [62]. EGCG also has potent metal-chelating properties through the presence of the pyrogallol group in its structure, which enable its binding to transition metal ions and function as a proactive antioxidant for cancer treatment [63]. Furthermore, studies have shown that EGCG can prevent cancer progression by affecting several signalling pathways and processes, such as DNA hypermethylation, angiogenesis, apoptosis, and NF-kB activation [64].

It was suggested that the daily consumption of green tea can aid in the treatment and prevention of cancer [65]. However, clinical trials have shown a weaker link between green tea intake and its effectiveness in fighting cancer [19,20,66]. These contradictory observations maybe attributed to the poor bioavailability of EGCG [64]. In fact, it has been shown that systemic levels of EGCG in humans resulting from the oral consumption of green tea catechins are 5 to 50 times lower than the concentrations needed to induce biological effects in vitro [67]. Furthermore, the degradation of EGCG in alkaline and neutral conditions has been attributed to the deprotonation of its hydroxyl functional groups [68]. In addition, the low bioavailability of catechins maybe contributed to their relatively high molecular weight and the presence of hydrogen-donating hydroxyl groups. Furthermore, the efflux effect of multidrug-resistant proteins on green tea catechins is another reason for their poor bioavailability [69].

Loading EGCG into NPs was used to improve its bioavailability. In this regard, Siddiqi et al. (2009) compared the anticancer effects of the encapsulated and free EGCG on PC3. The study found that encapsulated EGCG was more effective, with an IC_50_ value of 3.74 µmol/L, compared to 43.6 µmol/L for free EGCG. Using flow cytometry techniques, it was found that only a concentration of 2.74 µmol/L of capsulated EGCG was needed to induce apoptosis in around 70% of the cells, while a higher concentration of free EGCG (40 µmol/L) was needed to achieve similar levels of apoptosis. The study also showed that encapsulated EGCG reduced prostate tumour sizes in mouse cancer models more effectively than free EGCG [30]. Similarly, Shafiei et al. (2015) observed the higher cytotoxicity of EGCG loaded into inorganic NPs compared with free EGCG. The positive zeta potential value of the inorganic NPs was suggested to assist in their attachment to the negatively charged cancer cells. The authors also reported increased levels of apoptosis in the cells treated with EGCG-inorganic NPs [32]. Another study described the encapsulation of EGCG in a gum Arabic and maltodextrin matrix, which induced higher cytotoxic effects in androgen-insensitive Du145 cells than free EGCG, as determined via the clonogenic assay [31]. To avoid the degradation of EGCG in the gastric environment, Khan et al. (2014) developed a treatment for oral delivery by encapsulating EGCG in chitosan NPs and showed its controlled release under neutral pH conditions [34]. The oral administration of the chitosan-EGCG NPs to athymic nude mice grafted with human prostate cancer cells (22Rν1) subcutaneously led to a significant reduction in the tumour volumes. The tumour size in mice treated with a dose of 6 mg/kg body weight at day 32 was 216 mm^3^. On the same day, the tumour volumes measured for the control group and those treated with free EGCG were 1200 and 514 mm^3^, respectively. Immunoblots showed that the treatment with chitosan-EGCG NPs resulted in the initiation of apoptosis via the cleavage of PARP, the overexpression of Bax, the downregulation of Bcl-2, and the activation of caspases [34]. Furthermore, another study reported the encapsulation of EGCG with lipid NPs, which was optimized by varying the lipid content and the amount of co-lipid using an emulsion solvent evaporation method. The study showed that encapsulated EGCG was around four times more cytotoxic to Du145 cells compared to free EGCG, and staining the cells with Hoechst 33,342 dye indicated around a 25% increase in the percentage of apoptotic bodies with the encapsulated EGCG treatments [35].

Other studies utilized prostate cells targeting agents to improve the activity of EGCG-based NPs against PC. Sanna et al. (2011) loaded EGCG into polymeric NPs made of poly-e-caprolactone (PCL) and poly-d,l-lactide-co-glycolide/polyethyleneglycol (PLGA-PEG) and introduced a targeting moiety on the NPs by conjugating the NPs with pseudomimetic dipeptide N-[N-[(S)-1,3-dicarboxypropyl]carbamoyl]-(S)-lysine, which is a PSMA ligand. The in vitro cytotoxicity results exhibited greater antiproliferative activity for the targeting NPs against the PSMA-positive androgen-sensitive human prostate cancer cells (LNCaP) after 48 and 72 h of incubation when compared with the non-targeting NPs [29]. Another interesting synthesis technique reported by Chu and co-workers led to the formulation of PEG-Gelatine-based NPs loaded with EGCG and curcumin to evaluate whether these phytochemicals had synergistic effects [36]. The NPs were conjugated with hyaluronic acid (HA) and fucoidan, which served as CD44 and P-selectin targeting agents, respectively. CD44 is a highly expressed surface protein in solid tumours [70], while P-selectin is a tumour vasculature biomarker [71]. These NPs are pH-sensitive and only undergo morphological changes in acidic pH conditions, which are comparable to that of the tumour environment. This alteration characteristic represents a form of smart release where the NPs release their therapeutic loads with great precision in the tumour acidic site. Increased cytotoxic effects were demonstrated in PC3 cells for treatments with EGCG- and curcumin-loaded NPs compared to the treatments with free EGCG and curcumin [36]. The same group also reported the incorporation of EGCG (without curcumin) in iron oxide nanoparticles (FeO NPs) using PLGA as a colloidal carrier [37]. However, the IC_50_ values of the PLGA-EGCG NPs observed on PC3 cells were higher than the IC_50_ values observed when EGCG was used in combination with curcumin in their previous study, which clearly indicates the synergistic effects of both EGCG and curcumin [37].

EGCG has been found to have affinity for 67-kDa laminin receptor (67LR), which is overexpressed in several cancers, including PC [72]. Taking advantage of this feature, Shukla et al. (2012) used EGCG and radioactive isotope Au^198^ to synthesize radioactively labelled gold nanoparticles (EGCG-Au^198^NPs) [33]. The study evaluated the ability of EGCG to increase the targeting effect of the NPs in 67LR-overexpressing PC3 cells. Dark field microscopy confirmed the internalization of the EGCG-Au^198^NPs in PC3 cells, which could be inhibited by blocking 67LR with a 67LR-blocking antibody. In vivo studies using SCID mice carrying PC3-grafted cells reported in the same study showed a four-fold reduction in the tumour volume in the group treated with EGCG-Au^198^NPs compared to the control groups (treatments with saline and EGCG alone) [33].

### 2.2. Noscapine Based NPs

Noscapine (narcotine) is an alkaloid derived from opium (*Papaver somniferum*). It is a major opioid component of opium, accounting for approximately 10% of its total opioid content, second only to morphine [73]. Although it was originally identified as an antitussive agent, the anticancer properties of noscapine were reported due to its ability to act as a tubulin inhibitor [74]. Unlike other antimicrotubule agents such as taxanes, colchicine, or vinca alkaloids, noscapine can induce cancer cell death with minimal side effects [75]. However, noscapine is a lipophilic compound with average aqueous solubility, making it susceptible to hepatic metabolism. So, despite its potent cytotoxic activity and low toxicity, noscapine is characterized by poor oral bioavailability with a high oral effective dose of ED_50_ 300–600 mg/Kg [76].

Abdalla et al. (2011) developed a triple-conjugated system composed of FeO NPs, a human-type ATF (hATF), uPA, and a fluorescent dye (cy5.5) to deliver noscapine to PC cells. The authors confirmed the binding of the NPs to PC3 cells using Prussian blue staining. Additionally, the crystal violet assay showed a 6-fold increase in cytotoxicity for the loaded noscapine compared to the unmodified compound [41].

### 2.3. Curcumin Based NPs

Curcumin, also known as diferuloylmethane, is a major yellow polyphenolic compound found in the spice turmeric (Curcuma longa; Family: Zingiberaceae). It is widely used as a food additive and has been investigated for its potent antioxidant, anti-inflammatory, and anticancer activities [77,78]. Curcumin exerts its anticancer action by inducing cell death via apoptosis and silencing various cellular signalling pathways that contribute to tumour invasion [79,80]. However, the poor hydrophilicity of curcumin limits its use in medicine due to its inadequate bioavailability and chemical stability in vivo [81]. Additionally, curcumin has poor cellular uptake due to its hydrophobicity, which facilitates its interaction with the cellular membrane rather than the cytoplasm [82]. To overcome these limitations, researchers have sought to load curcumin into NPs to enhance its bioavailability.

Using a wet-milling technique, NPs made entirely of curcumin were produced and reported. The size of these NPs ranged between 34 to 359 nm [38]. Interestingly, in PC3 cells, as shown using MTT assays, the IC_50_ value of the free curcumin was two times higher than the IC_50_ value of the curcumin NPs. The authors suggested that this was due to the increased cellular uptake of the curcumin NPs [38]. However, other reports have highlighted the importance of the NPs’ size in cellular uptake, with a suggested size limit of 200 nm [83]. Thus, reducing the size of the curcumin NPs in this study may have led to higher cytotoxicity.

In another study, curcumin was loaded into liposomes with the encapsulation efficiency varying based of the type of lipid used [39]. The treatment of LNCaP cells with 5 to 10 µM of curcumin loaded into liposomes made of dimyristoyl phosphatidyl choline (DMPC) resulted in 70–80% cell death, while free curcumin only achieved similar effects at a much higher dose of 50 µM [39]. In a separate study, Narayanan et al. (2009) loaded curcumin and resveratrol (a phytoalexin from grapes) into separate liposomes and found that the co-administration of these two liposomal NPs had enhanced bioavailability in the serum and prostate tissue in mouse models compared to the free compounds or the naked liposomes [40]. This co-treatment also reduced the prostate weight and the number of adenocarcinomas accompanied by histological changes in prostate-specific PTEN knockout mice. The authors suggested that resveratrol’s affinity towards serum albumin enhanced the bioavailability of liposomal curcumin in the blood, thereby increasing its cytotoxic effects [40].

### 2.4. Berberine-Based NPs

Berberine, also known as “Natural Yellow 18”, is an isoquinoline quaternary alkaloid that has been shown to be present in several medicinal plants of different families, including Berberidaceae, Ranunculaceae, and Rutaceae [84]. The anticancer effects of berberine are well documented in both in vitro and in vivo studies. These effects are related to its regulation of cancer-causing genes and the suppression of different enzymes overexpressed in cancer tissues such as N-acetyltransferase, cyclooxygenase-2, and topoisomerase [85,86,87]. Additionally, berberine treatment has been shown to cause an increase in ROS production and mitochondrial transmembrane potential and stimulate nuclear factor-kappa B. This may explain its apoptotic effects [88]. However, berberine has an absolute bioavailability of less than 1.0%, which hinders its application in its free form as an anticancer treatment [89].

Shen and co-workers increased the solubility of berberine by 300% with a 30-fold increase in its pharmacokinetics by encapsulating it in micelles composed of 1,2-distearoyl-sn-glycero-3-phosphoethanolamine-N-[methoxy(polyethyleneglycol)-2000] (PEG-PE) and D-α-tocopheryl polyethylene glycol 1000 succinate (TPGS). Due to its hydrophobic nature, the incorporation of TPGS increased the loading and stability of berberine. This micelle formulation was three times more toxic to PC cells than the free berberine treatment [42].

### 2.5. Eupatorin-Based NPs

The flavonoid eupatorin also displays low pharmacokinetics, which contributes to its poor systemic delivery, short half-life in plasma, insolubility in aqueous media, poor bioavailability and oral absorption, low cellular uptake, and susceptibility to the first-pass effect [90]. One study reported the entrapment of eupatorin in polymeric NPs composed of PEG and PLGA and also then magnetised with FeO NPs [43]. The loaded eupatorin showed sustained release from the polymeric NPs and increased cytotoxicity against Du145 and LNCaP cell lines compared to the free eupatorin. Cell cycle analysis showed that eupatorin-loaded NPs increased the sub-G1 cell population in both prostate cancer cell lines, whereas the free eupatorin increased the G2-M interphase in these cells [43]. This indicates that the eupatorin-loaded NPs can stimulate DNA fragmentation, leading to apoptotic death.

### 2.6. Plant-Extract-Based NPs

Due to their high surface-to-volume ratio, nanosized structures exhibit different physicochemical properties compared to bulk structures [91]. Consequently, NPs demonstrate greater activity in various biological applications [92]. Therefore, reducing the size of plant extracts via conventional methods, such as precipitation, can enhance the bioactivity of the phytochemicals present in plant extracts. In most cases, this is accomplished by using plants with known folk medicine uses or those which have been reported to have biological activity. Cherian and co-workers (2015) reported the synthesis of *Baliospermum montanum* NPs (an ingredient of the ayurvedic herbal formulation, with known anticancer properties) via the precipitation method using the aqueous and ethanolic extracts of the plant [44]. The NPs derived from the two extracts exhibited higher cytotoxicity than the crude extract in PC3 cells. However, NPs from the ethanolic extract caused higher levels of cytotoxicity. According to the authors, the reason for this was the presence of anticancer steroids, triterpinoids, and ester compounds in the ethanolic extract, as shown in the FTIR spectrum, which were likely incorporated into the NPs during synthesis [44]. The same authors also reported the synthesis of precipitation-derived NPs using the ethanolic extract of *Leucas aspera*, which is another component of the ayurvedic herbal formulation. Significant cytotoxicity in PC3 cells was also observed after the *L. aspera*-NP treatment, with high levels of NP uptake detected in the treated cells compared to the positive control [45].

### 2.7. Plant-Derived Metallic NPs

A study by Bello et al. (2017) demonstrated the biosynthesis of AgNPs from *Hyphaene thebaica* (Doum plant) [46], whose phytochemicals are known to have anticancer activity [93]. The biogenic AgNPs displayed dose-dependent growth inhibition in PC3 cells [46]. A similar study by the authors showed the antiproliferation activity of AgNPs biosynthesized from the aqueous extract of *Guiera senegalensis*, a commonly used medicinal plant in Africa with reported anticancer activity [94]. These AgNPs displayed anticancer activity in PC3 cells [47]. Indeed, these AgNPs showed selectivity towards PC3 cells compared to other cancer cells lines such as MCF7 (breast cancer cells) and HepG2 (hepatic cancer cells), with IC_50_ values of 23.48, 29.25, and 33.25 μg/mL, respectively. The Chinese *Cornus officinalis*, which is used in Chinese herbal medicine, is another tree known for its antitumour activity [95]. Quasi-spherical AgNPs were biosynthesized from the aqueous fruit extract of this tree within 4 h of incubation with a silver salt solution. The FTIR data indicated that flavonoids and/or anthocyanins of *C. officinalis* play a role in the synthesis and stabilization of AgNPs. An IC_50_ of 25.54 μg/mL was determined for these NPs against PC3 cells using MTT assays, with no observed cytotoxicity for the extract [48]. The IC_50_ values of biogenic AgNPs formulated by Bethu et al. (2018) using *Rhynchosia suaveolens* were, respectively, determined to be 4.35 and 7.72 μg/mL in DU145 and PC3 cells. The authors claimed that the flavonoids and proteins contained in the extract were involved in the NPs’ biosynthesis [49]. The flavonoid and protein contents of *Indigofera hisruta* were also suggested to be the reducing and stabilizing agents that allowed for the production of stable AgNPs in another study. An IC_50_ of 68.5 μg/mL was determined for these plant-derived AgNPs against PC3 cells using MTT assays [50]. Zhang and co-workers used the Chinese herb *Salvia miltiorrhiza* to synthesize AgNPs with antiproliferative activity against LNCaP cells. Acridine orange (AO) and propidium iodide (PI) dye staining, as well as the TUNEL assay, demonstrated the apoptotic activity of the NPs. The induction of apoptosis via these AgNPs was also confirmed by an increase in the production of ROS [51]. Another study used the butanol soluble fraction of *Pinus roxburghii* pine needles to produce spherical AgNPs [52]. The choice of this organic solvent fraction was motivated by its previously demonstrated anticancer properties. With IC_50_ values of 56.27 ± 1.17 μg/mL, the synthesized AgNPs also exhibited potent anticancer activity against PC3 cells in comparison to the butanol extract, which had an IC_50_ value of 233.4 ± 1.12 μg/mL. Again, the increased levels of apoptosis observed following AgNPs’ treatment was accompanied by an increase in ROS production, affecting mitochondrial function, showing an increase in the cell population in the sub-G1 phase, caspase-3 activation, and PARP-1 cleavage [52]. Furthermore, ethanolic extracts of the fruit and leaves of the medicinal plant *Annona muricata* were used to produce AgNPs with higher cytotoxicity against PC3 (IC_50_ values below 50 μg/mL) than the positive control (5-fluorouracil; IC_50_ = 235.9 μg/mL) [53]. Another study producing AgNPs from *Perilla frutescens* leaf extract reported an IC_50_ value of 24.33 μg/mL against LNCaP cells. The phase-contrast microscopy images of cells treated with these AgNPs showed morphological changes pertaining to apoptotic induction such as cell shrinkage, membrane disturbance, and the condensation of chromatin [54].

Zinc nanoparticles (ZnNPs) fabricated using the aqueous leaf extract of *Hyssopus officinalis* showed significantly low IC_50_ values on PC3 (5.0 µg/mL). These NPs also activated apoptosis in cells in a dose-dependent manner [56]. Copper oxide nanoparticles (CuO NPs) were also biosynthesized using the highly nutritious and medicinal plant *Rhus punjabensis*. While the CuO NPs displayed anticancer activity against PC3 as demonstrated by the sulforhodamine B colorimetric assay, the anticancer activity of the plant extract was higher. Furthermore, the CuO NPs showed inhibition to NF-κB signalling, which further highlights their application in fighting cancer [57]. Gold nanoparticles (AuNPs) were also biosynthesized from different extracts of *Euterpe oleraceae* (Acai berry) and *Sambucus nigra* (Elderberry), which showed increased cytotoxic effects against PC3 cells in comparison to the extracts [55].

### 2.8. Loading Plant Extracts into NPs

Other approaches were reported when describing the anticancer effects of plant-extract-based NPs such as the direct loading of plant materials into NPs. These NPs are mostly polymeric in nature, which also enhance their overall biocompatibility [96]. These polymeric NPs would also allow the controlled release of phytochemicals to enhance their anticancer action [97]. For example, the anticancer activity of *Nigella sativa* motivated Dawaba and Dawaba (2018) to load this plant’s essential oil into NPs composed of chitosan connected to cinnamic acid and benzoic acid in different ratios. Around 90% of the essential oil was released at pH 7.4, indicating the ability of these NPs to release the essential oil into the bloodstream and then to the target site. Further, the MTT results showed significant antiproliferation activity against the PC3 cells (17.95 ± 0.82 µM) as compared to the free oil (43.56 ± 1.95 µM). This activity was attributed to the loaded essential oil as the naked NPs did not exhibit any cytotoxicity [58]. Another interesting study by Ribeiro et al. (2020) loaded *Uncaria tomentosa* extract, which has known anticancer activity, into different polymeric NPs (PCL and PLGA) [59]. The High Performance Liquid Chromatography analysis for the total alkaloids showed higher drug loading in PLGA NPs than in PCL NPs. This is because of the acidic groups present in PLGA, which facilitate stronger interaction with the amino groups of the alkaloids, as suggested by the authors. The in vitro assay showed higher cytotoxicity for PLGA NPs than PCL NPs against DU145 cells [59].

### 2.9. Reported Toxicity of the Reviewed NPs

The reason for incorporating biological materials such as plant extracts in the synthesis of NPs is mainly for the production of biocompatible NPs intended for safe therapeutic applications [98]. However, consideration should be given to the possible side effects of the unknown phytochemicals present in the extracts [99], which may play a role in the synthesis and stabilization of the NPs. The toxic effect of metal ions, especially silver ions [100], is another consideration. Therefore, when reporting on plant-derived NPs, it is vital to evaluate their possible toxicity. Table 2 shows a summary of toxicity evaluations reported from the studies reviewed above. Only a few reports studied the effects of the “green” NPs in vivo, with most of the studies showing the effect of these NPs on both cancerous and normal cells.

## 3. Conclusions

Several plant extracts have been used to biosynthesize NPs. This is attributed to the different phytochemicals found in these extracts that contain reducing functional chemical groups. The role of the plant extracts or the phytochemicals in these NPs is variable. Some act as reducing agents, while others function as stabilizing agents. In some instances, the resultant NPs may act as carriers for the phytochemicals of anticancer properties. Some phytochemicals may also enhance the targeting effect of the NPs. A number of these NPs have shown strong anticancer properties against PC cell lines. Most of the studies measured the in vitro activity of the NPs by evaluating them on several PC cell lines such as PC3, Du145, and LNCaP. Meanwhile, some reports used in vivo animal cancer models consisting of cell grafts of similar cell lines to determine the anticancer activity of these plant-derived NPs. Studies aimed at evaluating the anticancer effects of these NPs also demonstrated the ability of the NPs to activate apoptosis by upregulating apoptotic markers, affecting cell cycles, or by altering cell morphologies. Plant extracts used in the studies were mainly aqueous extracts, while a few reports used organic solvents extracts. The latter targeted specific anticancer natural products that might not be found in aqueous extracts. Therefore, it is vital to focus on isolating the active phytochemicals first if the use of organic solvents is needed to abide with the green chemistry guidelines. Moreover, several reports evaluated the safety of green NPs by comparing their toxicity on normal cells and cancerous cell lines or by performing blood evaluations and measuring the weights of mice in the case of in vivo studies. These evaluations should be encouraged to verify the safety of these NPs for clinical use.

## Figures and Tables

**Table 1:** Summary of the NPs with activity against PC.

NPs Type	NPs Composition	Shape	Size (nm)	Size Determination Technique	Encapsulation Efficiency %	Anticancer Effect	References
Epigallocatehin Gallate (EGCG)-based NPs	poly-d,l-lactide-co-glycolide/polyethyleneglycol (PLGA-PEG) NPs conjugated with anti PSMA	Spherical	80.53 ± 15.0	Scanning electron microscopy (SEM)	9.61 ± 0.7	Increased cytotoxicity activity against androgen-sensitive human prostate cancer cell line (LNCaP).	[29]
	Polylactic acid-PEG NPs	Not reported (NR)	260	Dynamic light scattering (DLS)	NR	Increased cytotoxicity against androgen-insensitive human prostate cancer cells (PC3); induction of apoptosis; antiangiogenesis effect; prostate tumour size reduction in mice.	[30]
	Gum Arabic–maltodextrin matrix	Spherical	120	DLS	85 ± 3	Increased cytotoxicity against androgen-insensitive human prostate cancer cells (Du145).	[31]
	Inorganic NPs	Nano-flakes of hexagonal shapes	10	Transmission electron microscopy (TEM)	NR	Increased cytotoxicity against PC3; induction of apoptosis.	[32]
	Gold nanoparticles (AuNPs)	Spherical	40–55	TEM	NR	Increased cytotoxicity against PC3 in vivo.	[33]
	Chitosan NPs	Spherical	150–200	TEM	10%	Increased cytotoxicity against human prostate cancer cells (22R*ν*1) implanted in mice.	[34]
	Lipid NPs (glycerol monostearate/stearic acid/soya lecithin)	NR	157	DLS	67.2 ± 4.5	Increased cytotoxicity against Du145 cells; apoptosis induction.	[35]
	PEG-Gelatine NPs conjugated with hyaluronic acid (HA) and fucoidan. EGCG was loaded in combination with curcumin	Spherical	197.73 ± 18.53	TEM	46.01 ± 1.96 (EGCG), 67.76 ± 6.67 (curcumin)	Increased cytotoxicity against Luc PC3 both in vitro and in vivo.	[36]
	PLGA-PEG-Gelatine NPs conjugated with HA and fucoidan.	Spherical	217.19 ± 11.37	DLS	19.67 ± 2.48	Increased cytotoxicity against PC3 both in vitro and in vivo; upregulation of caspases; reduction in G0/G1 and increase in S-phase population.	[37]
Curcumin-based NPs	Curcumin NPs	NR	34–359.4	SEM	Not applicable (N/A)	Increased cytotoxicity against PC3.	[38]
	Liposome NPs	NR	100–150	DLS	0.125 mg/ml	Increased cytotoxicity against LNCaP.	[39]
	Liposome NPs	NR	NR	NR	NR	Reduction in the prostate weight and the number of adenocarcinomas in prostate-specific PTEN knockout mice; apoptosis induction; increase in the pre-G1 population; downregulation of p-Akt, cyclin D1, AR, and mTOR from PTEN-Cap8 cells.	[40]
Noscapine-based NPs	Iron oxide nanoparticles (FeO NPs) loaded with urokinase-type plasminogen activator(uPA) targeting moiety (human-type ATF (hATF) and fluorescent dye (cy5.5)	Spherical	35.62 ± 4.1	DLS	88.2 ± 2.3	Increased cytotoxicity and targeting towards PC3 cells.	[41]
Berberine-based NPs	Mixed polymeric phospholipid micelles of 1,2-distearoyl-sn-glycero-3-phosphoethanolamine-N-[methoxy(polyethyleneglycol)-2000] (PEG-PE) and D-α-tocopheryl polyethylene glycol 1000 succinate (TPGS)	Micelles	24.1 ± 1.8	DLS	95.7 ± 3.6	Increased cytotoxicity against PC3 and LNCaP cells.	[42]
Eupatorin-based NPs	Eupatorin loaded on copolymer of magnetic PEG and PLGA	Spherical	58.5 ± 4	DLS	90.99 ± 2.1	Antiproliferation activity against Du145 and LNCaP; apoptosis induction; activation of sub-G1-phase population; upregulation of caspase 3 and Bax/Bcl-2 ratio.	[43]
Plant-extract-based NPs	*Baliospermum montanum-*based NPs via precipitation method	Irregular	211.3 and 233 for NPs from the aqueous and ethanolic extracts, respectively	DLS	N/A	Increased cytotoxicity against PC3 cells.	[44]
	*Leucas aspera-*based NPs via precipitation method	Spherical	200–400	DLS	N/A	Increased cytotoxicity against PC3 cells.	[45]
	Silver nanoparticles (AgNPs) using *Hyphaene thebaica* aqueous extract	Spherical	20	TEM	N/A	Cytotoxic activity against PC3.	[46]
	AgNPs using *Guiera senegalensis* aqueous extract	Spherical	50	TEM	N/A	Cytotoxic activity against PC3.	[47]
	AgNPs using *Cornus Officinalis* fruit aqueous extract	Quasi-spherical	11.7	TEM	N/A	Cytotoxic activity against PC3.	[48]
	AgNPs using aqueous leaf extract of *Rhynchosia suaveolens*	Spherical	10–30	TEM	N/A	Selective cytotoxic activity against PC3 and DU145 cells.	[49]
	AgNPs using aqueous leaf extract of *Indigofera hisruta*	Spherical	5–10	TEM	N/A	Selective cytotoxic activity against PC3 cells.	[50]
	AgNPs from *Salvia miltiorrhiza* leaf aqueous extract	Different shapes	12–80	TEM	N/A	Cytotoxic activity against LNCaP; apoptosis induction. Increased Reactive oxygen species (ROS) production.	[51]
	AgNPs from the butanol fraction of *Pinus roxburghii* needles.	Spherical	80	TEM	N/A	Increased cytotoxicity against PC3; apoptosis induction. Increased ROS production and mitochondrial dysfunction; increased cells in the sub-G1 phase.	[52]
	AgNPs from the ethanolic fruits and leaves extracts of *Annona muricata.*	NR	NR	NR	N/A	Increased and selective cytotoxic activity against PC3.	[53]
	AgNPs from the aqueous leaf extract of *Perilla frutescens*	Different shapes	20–50	TEM	N/A	Cytotoxic activity against LNCaP.	[54]
	AuNPs from different solvent extracts of *Euterpe oleraceae* and *Sambucus nigra*	Different shapes	Variable according to the type of extract used	DLS/TEM	N/A	Cytotoxic activity against PC3.	[55]
	Zinc oxide NPs (ZnO NPs) from the aqueous leaf extract of *Hyssopus officinalis*	NR	NR	NR	N/A	Cytotoxic activity against PC3; apoptosis induction.	[56]
	Copper oxide nanoparticles (CuO NPs) from the aqueous leaf extract of *Rhus punjabensis*.	Spherical	31.27	SEM	N/A	Cytotoxic activity against PC3; inhibition of NF-κB signalling.	[57]
	Loaded *Nigella sativa* essential oil in chitosan NPs connected to different ratios of benzoic and cinnamic acids.	Spherical	341	TEM	98	Increased cytotoxic activity against PC3.	[58]
	PLGA and poly-e-caprolactone (PCL) NPs loaded with *Uncaria tomentosa* extract	Spherical	247.3 ± 9.9 for PCL-based NPs, and 107.4 ± 3.0 for PLGA-based NPs	DLS	81.6 ± 0.7% for PCL-based NPs and 64.6 ± 2.0% for the PLGA-based NPs	Cytotoxic activity against LNCaP and Du145.	[59]

**Table 2:** Toxicity evaluation reported for the plant-derived NPs affecting prostate cancer.

Type of NPs	Toxicity Summary	References
EGCG NPs	EGCG polymeric NPs showed selective toxicity against LNCaP, while no cytotoxicity was exhibited against human umbilical vein endothelial cells (HUVECs).	[29]
Curcumin NPs	The curcumin NPs showed selective cytotoxicity against PC3 (IC_50_ = 121.92 µM) as compared to the mammalian cell line (HEK 293) (IC_50_ = 292.88 µM).The curcumin NPs showed comparable haemolysis % to the parent curcumin with concentrations up to 600 µM.	[38]
	A seven-week administration of liposomal curcumin, liposomal resveratrol, and their co-administration (at a dosage of 50 mg/kg and 25 mg/kg, respectively) did not cause any significant toxicity or weight changes in mice.	[40]
Berberine NPs	The micelles loaded with berberine showed higher hemocompatibility as compared to the free berberine.	[42]
Plumbagin NPs	The plumbagin NPs were less toxic to normal cells as compared to the crude extract. The NPs also showed high blood biocompatibility in vivo.	[101]
Eupatorin NPs	The polymeric NPs loaded with eupatorin exhibited selective cytotoxicity against Du145 and LNCaP cell lines, while not exhibiting any cytotoxic effect on HUVECs.	[43]
Plant extract NPs	The B. montanum extract NPs were cytotoxic against PC3 cells but did not show growth inhibition against normal mouse embryonic fibroblasts (NIH3T3). In addition, mixing the extract NPs with blood did not exhibit significant haemolysis.	[44]
	The L. aspera extract NPs were blood biocompatible, as no agglomeration of different blood cells was detected at a concentration of 0.25 mg/mL.	[45]
	The AgNPs from R. suaveolens exhibited 16 times higher toxicity against PC3 and DU145 cancer cells compared to Chinese hamster ovary (CHO) cells.	[49]
	The AgNPs derived from I. hisruta did not exhibit cytotoxicity against CHO cells at the highest concentration tested, in contrast to their cytotoxic effect against PC3 cells.	[50]
	The AgNPs derived from P. roxburghii demonstrated selective antiproliferation activity against two cancer cell lines, while showing no activity on two normal human cell lines, including normal human breast epithelial cells (fR2) and human peripheral blood lymphocytes (PBL).	[52]
	The AgNPs synthesized from the fruits and leaves of A. muricata demonstrated a selective index of 7.8 and 2.26, respectively, against the PC3 cell line as compared to the normal prostate epithelium (PNT1A) cell line.	[53]

## Data Availability

Not applicable.

## List of Abbreviations

22Rν1	Human prostate cancer cells
67LR	67-kDa laminin receptors
AO	Acridine orange dye
AuNPs	Gold nanoparticles
CHO	Chinese hamster ovary cells
CuO NPs	Copper oxide nanoparticles
DLS	Dynamic light scattering
DMPC	Dimyristoyl phosphatidyl choline
Du145	Androgen-insensitive human prostate cancer cell line
FeO NPs	Iron oxide nanoparticles
fR2	Normal human breast epithelial cells
HA	Hyaluronic acid
hATF	Human-type ATF
HepG2	Human hepatic cancer cells
HUVECs	Human umbilical vein endothelial cells
LNCaP	Androgen-sensitive human prostate cancer cell line
MCF7	Human breast cancer cells
MTT	Tetrazolium-dye-based assay
N/A	Not applicable
NIH3T3	Normal mouse embryonic fibroblast
NR	Not reported
PBL	Human peripheral blood lymphocytes
PC	Prostate cancer
PC3	Androgen-insensitive human prostate cancer cells
PCL	Poly-e-caprolactone
PEG	Polyethyleneglycol
PEG-PE	1,2-distearoyl-sn-glycero-3-phosphoethanolamine-N-[methoxy(polyethyleneglycol)-2000]
PI	Propidium iodide dye
PLGA	Poly-d,l-lactide-co-glycolide
PNT1A	Normal prostate epithelium cells
PSMA	Prostate-specific membrane antigen
ROS	Reactive oxygen species
SEM	Scanning electron microscopy
TEM	Transmission electron microscopy
TPGS	D-α-tocopheryl polyethylene glycol 1000 succinate
uPA	Urokinase-type plasminogen activator
ZnO NPs	Zinc oxide nanoparticles
